# Development of an online supportive treatment module for moral injury in military veterans and police officers

**DOI:** 10.3389/fpsyt.2022.890858

**Published:** 2022-09-02

**Authors:** F. Jackie June ter Heide, Mariëlle L. de Goede, Sanne van Dam, Stijn Ekkers

**Affiliations:** ARQ Centrum'45, Oegstgeest, Netherlands

**Keywords:** moral injury, police officers, military veterans, e-health, online treatment

## Abstract

**Background:**

Military members and police officers often operate in high stakes situations and under high levels of physical and psychological stress. Consequently, they may be confronted with morally injurious experiences and develop moral injury. Most treatments for moral injury are cognitive-behavioral, face-to-face treatments, which may be supported by online interventions. Online interventions have shown promise in the treatment of trauma-related psychopathology, but few such interventions for moral injury yet exist.

**Objective:**

To develop and conduct a preliminary evaluation of an online treatment module for moral injury in treatment-seeking military veterans and police officers, to be used in conjunction with regular face-to-face treatment.

**Method:**

An online module was developed based on the moral injury literature, using elements from seven existing treatments. A preliminary evaluation was conducted using both quantitative and qualitative methods, and focusing on perceived feasibility, acceptability and engagement of the module, as well as potential benefits and harms. The concept module was evaluated by 15 assessors, including patient representatives, multidisciplinary caregivers and experts.

**Results:**

The module was rated favorably, with mean evaluation scores ranging from 7.9 to 8.8 on a 10-point scale. Several suggestions for improvement were made, especially concerning privacy issues, safety instructions, patient-therapist collaborations, and role plays, and the module was adapted accordingly.

**Conclusion:**

Using input from literature, patient representatives and experts, we developed an online treatment module for moral injury in military veterans and police officers, to be used in conjunction with face-to-face therapy. Acceptability and feasibility will be further examined in a future pilot study.

## Introduction

The concept of moral injury refers to the lasting and multidimensional impact of perpetrating, failing to prevent or witnessing acts that transgress deeply held moral expectations and beliefs (1). Intentionally harming a civilian during armed conflict, failing to save a child from a fire, or standing by as a colleague mistreats a prisoner are examples of such acts. Involvement in morally transgressive acts may lead to moral injury, especially when it occurs in high stakes situations (2), where those involved risk death, serious injury or sexual violence (3). While the moral injury concept has been predominantly developed and studied in military populations, it may also apply to other populations exposed to occupational trauma, especially police officers (4, 5). Like military members, police officers are trained to serve and protect, meaning they may use legitimate, but not excessive, force, and must act to save civilians from serious harm. Given that such tasks may be performed under high levels of physical and psychological stress, both military members and police officers are at risk of encountering potentially morally injurious events (PMIE's) and consequently, of developing moral injury.

Moral injury is a psychological, social and existential wound that has been found to be associated with the development of psychiatric problems and functional impairment, including posttraumatic stress disorder (PTSD), suicidal ideation and depressive symptoms (4, 6). Consequently, for some individuals who suffer from moral injury, psychological intervention may be necessary to increase their psychosocial wellbeing and quality of life. The development of interventions for alleviating moral injury is relatively in its infancy. Most psychological interventions for moral injury, including Adaptive Disclosure (AD) (7), Trauma-Informed Guilt Reduction Therapy (TrIGR) (8) and Acceptance and Commitment Therapy for Moral Injury (ACT-MI) (3) are based on cognitive-behavioral treatment (CBT) frameworks (9). Such frameworks commonly encourage patients to work on their treatment goals outside of the treatment room. In recent years, this is increasingly done through internet or e-health interventions. Internet-delivered CBT has shown promise in treating patients with posttraumatic stress disorder (PTSD) (10–12), including military veterans (13).

The term “internet interventions” may refer to a range of interventions, from complete internet-delivered treatments to digital treatment components such as online modules (14). Several such treatments or treatment components may be suitable for helping patients heal from moral injury. However, as far as we know, references to the use of internet interventions for moral injury are limited to a case study of the successful treatment of a service member using ACT-MI *via* telehealth (15), and a feasibility and acceptability study of an online therapy group for healthcare providers working during the COVID-19 pandemic (16). This implies that most treatments for patients with moral injury take place face-to-face. Supporting face-to-face treatment with internet interventions such as online modules may carry several benefits. Patients may work on their treatment goals from their own home and in their own time, keeping arousal low; patients may be provided with structured, accurate visual and written information that may be more easily processed or referred back to; patients and therapists may exchange information in a digitally secure environment; and insights derived from the internet intervention may then be shared in and inspire face-to-face treatment sessions.

In order to support the face-to-face treatment of patients with moral injury through internet interventions, we developed an online treatment module for moral injury in treatment-seeking military veterans and police officers in the Netherlands. Dutch military members are known to encounter PMIE's during peace-keeping missions, including being in the position of bystander, indirect effects of decisions and actions, and transgressive behavior (17). In a quarter of military veterans, this may lead to feelings of shame and guilt post-mission, which in turn is related to more severe depression and anger (17). During and after missions, Dutch military members and veterans may experience conflicting values as well as feelings of moral detachment and senselessness (18). Little research has been conducted on moral injury in Dutch police officers. Treatment-seeking Dutch police officers have been exposed to an average of 19.5 work-related potentially traumatic experiences, including PMIE's such as executing charges in which persons were injured, injury of a colleague, and failed cardiopulmonary resuscitation (19). Consequently, around 43% of treatment-seeking Dutch police officers meet symptom profiles of moral injury, with or without PTSD (20).

In this paper, we report on the development and preliminary evaluation of an online treatment module for treatment-seeking military veterans and police officers in the Netherlands. Aim of the development of this module was to make available an online intervention to support face-to-face treatment of military veterans and police officers with moral injury. Aim of the preliminary evaluation was to gain feedback on and improve the concept version of the module involving various stakeholders and experts. Our hypothesis was that the concept version of the module would be considered acceptable, feasible, engaging and not harmful, but might still be improved.

## Method

### Setting

The module was developed at ARQ Centrum'45, the Dutch national center for expert diagnostics and treatment of complex psychotrauma. The center offers tertiary care to trauma-exposed military veterans and police officers who have failed to benefit from or have relapsed after first-line treatment (21). Most of these patients meet criteria for PTSD according to DSM-5 and are routinely treated with treatments of choice following the Dutch treatment guidelines for PTSD (21): trauma-focused CBT [including prolonged exposure (PE), narrative exposure therapy (NET), and brief eclectic psychotherapy for PTSD (BEPP)] or eye movement desensitization and reprocessing (EMDR) therapy.

To support face-to-face treatment through e-health interventions, ARQ Centrum'45 uses an e-health platform called Minddistrict. Minddistrict offers online modules and diaries for patients that can be accessed through mobile devices. These modules are developed in collaboration with patient representatives and care providers. The modules are generally transdiagnostic, i.e., focused on complaints or symptoms rather than on diagnoses, and generally consist of written information, video material of experts and patient representatives, and online assignments. Assignments may be completed independently by the patient or may be shared with the therapist who then provides written feedback. All information exchanged through Minddistrict is secure, requiring a login and password.

### Development steps

Development was based on guidance for the development of complex interventions to improve health and healthcare (22), using the following steps (not necessarily in this order): (1) planning of the development process, (2) involving stakeholders, (3) bringing together a team, (4) reviewing published research evidence, (5) drawing on existing theories, (6) articulating program theory, (7) undertaking primary data collection, (8) understanding context, (9) paying attention to implementation, (10) designing and refining the intervention. Development ran from February 2021 till February 2022 and was chiefly conducted by a clinical psychologist, a social-psychiatric nurse and a communication expert, in consultation with Minddistrict, two patient representatives, an army chaplain and three therapists.

### Literature search

To decide on the content of the module, we conducted an APA PsycINFO search of peer-reviewed papers on psychotherapeutic treatments for moral injury using the search terms “moral injury AND (treatment OR therapy OR intervention OR manual).” The resulting evidence was limited. Case studies were found of ACT-MI (15), BEPP for Moral Trauma (BEPP-MT) (23), Cognitive Therapy (CT) (24, 25) and PE (24, 26). Pilot studies were found of AD (27) and Impact of Killing (IoK) (28–30). We excluded interventions intended primarily for delivery by chaplains or clergy, including Building Spiritual Strength (BSS) (31) and the Mental Health Clinician and Community Clergy Collaboration (32). In addition, we consulted the book *Addressing moral injury in clinical practice* (33), which provides an overview of treatment approaches for moral injury. Last, we searched for interventions for which detailed manuals or protocols had been published, which was the case for AD (7), TrIGR (8), and ACT-MI (3). AD (1, 7) is an integral cognitive-behavioral treatment of moral injury, involving eight steps: connection, preparation and psychoeducation, modified exposure, examination and integration of maladaptive beliefs, dialogue with a benevolent moral authority, reparation and forgiveness, fostering reconnection, and after-treatment planning. TrIGR (8) is a cognitive treatment that primarily focuses on the identification and appraisal of four domains of cognitive errors: hindsight bias, lack of justification, responsibility, and wrongdoing. ACT-MI (3) focuses on acceptance of moral pain (through interventions such as psychoeducation, defusion and mindfulness) and commitment to living a value-driven life (through interventions such as fostering forgiveness and compassion, and identifying and acting upon values).

In conclusion, we based the module on interventions described in the following treatments: ACT-MI, AD, BEPP-MT, CT, IoK, PE and TrIGR.

Integrating the literature, we concluded that the treatments include most if not all of the following interventions: (1) psychoeducation, (2) processing of morally injurious memories, (3) exploring maladaptive attributions, (4) mindfulness, (5) forgiveness, (6) reconnection, and (7) living according to important values. Those interventions may be perceived as addressing the three prominent domains that may be affected in moral injury: psychological (emotional, cognitive and behavioral), social/interpersonal, and spiritual/existential (1, 4).

### Module development

Based on the literature, we then developed a first version of the module. This version consisted of eight chapters with the following topics: module explanation, moral injury and PTSD, moral code and values (chapter 1), morally injurious experiences, moral emotions, moral pain and moral judgments (chapter 2), moral injury narrative, hotspot, prolonged exposure and EMDR (chapter 3), determining and exploring hindsight bias, lack of justification, responsibility and wrongdoing (chapter 4), a written, imaginary or actual dialogue with a benevolent moral authority (chapter 5), practicing mindfulness (chapter 6), the costs and benefits of forgiveness, and forgiving actions (chapter 7), determining values, and value-driven actions (chapter 8). The length of the module (eight chapters) was based on the average length of standardized treatments for moral injury, ranging from six (TrIGR) to ten (IoK) sessions. All chapters contained written information, video clips of patient representatives and professionals, and assignments.

#### Language

Following recommendations for therapist style and stance when working with morally injured patients, written information was carefully worded to be encouraging, supportive and non-judgmental (7). We took care to use inclusive language, for example by using case vignettes that alternately referred to military veterans and police officers, men and women, and persons with a western and non-western first name. In addition, we took language proficiency into account by limiting sentences to 15 words and avoiding use of the passive tense, in accordance with Dutch B1 language guidelines (34). Difficult words, such as abstract words and jargon, were avoided by using easier alternatives or by giving an explanation and/or illustrative example. To further improve readability, paragraphs were limited to 450 characters.

#### Video material

Video clips were filmed by a professional filmmaker. The final clips included descriptions of morally burdening experiences, moral emotions and cognitions, coping, reprocessing and reconnection, by a male military veteran and a female police officer who had both been in treatment for moral injury; a word of welcome and explanations of moral injury, prolonged exposure and EMDR, by a therapist; explanations on moral injury, morality, values, forgiveness and the work of a chaplain, by a military chaplain; and roleplays of a dialogue with a benevolent moral authority, exploring cognitive errors, and living a value-driven life, featuring a therapist and a patient played by a therapist. Care was taken that the descriptions of morally burdening experiences were specific enough to spark recognition but general enough not to upset patients. All video clips were pre-discussed and scripts were written of the roleplays in consultation with a military veteran. All clips were approved by those who featured in them before being included in the module. The two patient representatives signed informed consent forms for inclusion of the video clips in the module. They were debriefed after filming and received a gift coupon in recognition of their effort as well as reimbursement of their travel expenses. The military chaplain also received a gift coupon.

#### Assignments

Assignments were included that consist mostly of invitations to describe personal experiences of morally injurious events, moral injury symptoms, moral emotions and judgements, values and goals, and to examine moral judgments. To this end, spaces are provided where patients may insert text. In addition, patients are invited to watch video clips of patient representatives and experts, and psychoeducational videos of the Dutch societies for CBT and EMDR. Last, mindfulness exercises were inserted that are available through Minddistrict. Care was taken to include only assignments that might be performed at home, without the presence of a therapist, and asking patients to choose a good time and place and to note how they felt afterwards. At the end of each module chapter, assignments are saved and therapists receive an email alert to provide written feedback.

### Preliminary evaluation

#### Design

In order to improve the online module, we conducted a preliminary quantitative and qualitative assessment of module content, style, format and delivery. Evaluation focused on perceived feasibility, acceptability and engagement of the module, as well as potential value and benefits, harms and unintended consequences (22, 35).

#### Procedure

A questionnaire was made evaluating the content, format, style, delivery and perceived proceeds of the overall module, and the contents of the separate chapters. The questionnaire consisted of 13 quantitative items rated on a 10-point scale ranging from 1 (*very low*) to 10 (*very high*) (for example, “How would you rate the content of this chapter?”), as well as 21 open questions with room for comments and suggestions (for example, “Do you expect the module to be potentially harmful to users? If so, in what respect?”).

An email was sent to 15 stake holders and experts asking them to evaluate the module. Upon consent, they were provided with an online link to the module and to the questionnaire. Response was 100%. Four assessors provided only qualitative feedback. Evaluations were conducted by two patient representatives; one military chaplain; two researchers specializing in moral injury or e-health; and ten therapists from five treatment centers specializing in the treatment of military veterans, police officers and/or moral injury. As none of the patient representatives were currently in treatment and the evaluation did not concern medical research, no medical-ethical assessment was required.

#### Analysis

Descriptive statistics were calculated using SPSS version 23 for Windows. Answers to the open questions as well as additional written feedback sent by some assessors were inserted in Excel and analyzed following the General Inductive Approach for analyzing qualitative evaluation data (36). All text that was deemed relevant to the evaluation aims was labeled to identify themes. Next, in a second round of coding, some themes were merged to reduce overlap and redundancy among the codes. The resulting themes were subsequently combined under superordinate categories, which were based on the evaluation aims.

## Results

### Descriptive statistics

The module was rated very favorably, with the mean evaluation scores of various chapters and aspects ranging from 7.9 to 8.8. Module content was rated M = 8.8 (SD = 0.8, range 8–10), style M = 8.5 (SD = 0.8, range 7–10), format M = 8.5 (SD = 0.8, range 7–10), and delivery M = 8.2 (SD = 1.1, range 7–10). Perceived benefits for users were rated M = 8.5 (SD = 9.3, range 7–10).

### Qualitative analysis

Qualitative analysis revealed several themes related to various components of the module, which we organized across four categories: acceptability, feasibility, engagement, and unintended consequences (see Table 1).

**Table 1:** Sample quotations illustrating feedback categories and themes.

**Categories**	**Themes**	**Description**	**Sample quotation**
Acceptability	Suitability for the target population	The assessors believed the module is suitable for the target population. They appreciated the video examples provided by patient representatives, which were described as something patients would be able to relate to.	“The videos of the patient representatives are wonderful and illustrate the subject beautifully.”
	Confidentiality	Some assessors stressed the importance of explicitly addressing any confidentiality concerns patients may have, both at the start of the module and when exercises ask for the disclosure of personal experiences and thoughts.	“It might be good to also describe confidentiality/privacy in assignments where clients have to be vulnerable? (I) can imagine that there are distrustful clients who find it difficult to fill in their experiences on a website? Maybe suggest an alternative, for example: you can also write this down for yourself in a notebook.”
	Benefits	Assessors believed the module would most likely be beneficial to patients. Potential benefits they mentioned include an increased understanding of moral injury and better insight into how therapy and certain exercises can be helpful.	“(The module) would give me a lot of clarity and reassurance. To understand what it does to you, that it is not crazy what you are struggling with. It makes it easier to discuss things, also with your partner (…). This can lead to more understanding. I found these things difficult to explain myself, so this module is very clear and helpful.”
Feasibility	Clinical context	Many assessors stressed the importance of offering the module as blended treatment. They emphasized it would be best to conduct the module alongside in-person therapy, to maximize the potential of the module. Embedding the module within a clinical context was also perceived as helpful in case module topics were triggering.	“With the guidance of the practitioner, it is feasible. It could trigger a lot. There should be an option to contact your practitioner. Especially if your appointment is next week. Simply knowing that is possible is reassuring.”
Engagement	Clarity of information	Module information provided was generally evaluated as clear and complete. Particularly the videos of various experts and patient representatives were found to be illuminating and engaging. Some assessors offered suggestions for improving information.	“Good to see the concept of moral injury explained from different perspectives. Good differentiation between PTSD and moral injury, with the explanation of the patient representative. I think clients would be able to benefit a lot from that.”
	Overview of the module	Most assessors found the module clear and orderly structured, with a logical progression from one topic to the next. Some suggested the module would be easier to navigate if it had a more user-friendly menu to browse between chapters.	“It might be useful to have a navigation menu where you can browse back to all the different chapters. So if the patient wants to revisit the mindfulness chapter, this can be done with a single click in the navigation menu.”
	Suggestions for improvement	Assessors offered various practical suggestions for improving the module, including adding certain exercises and examples of how to work on recovery.	“Maybe explicitly state that (…), not only forgiveness and acceptance help, but also actively doing meaningful things? Achieving forgiveness not only through internal, mental processes, but also by actively doing things.”
Unintended consequences	Potentially triggering elements	Assessors believed risks associated with the module to be low. However, some potentially triggering elements were mentioned, including an exposure exercise and stories of moral injury shared by the patient representatives.	“I can imagine that this is a tough chapter for people who (still) have a lot of PTSD symptoms. Such as the question to write down memories. Though I expect that this would be considered at intake.”

#### Acceptability

All assessors deemed the module to be acceptable and to meet an important clinical need, as in the Netherlands treatments developed specifically for moral injury are limited. Several assessors noted that certain elements of the module, such as its focus on self-forgiveness and moral values, are not addressed in current treatments and therefore particularly valuable. Simultaneously, some expressed concern that these elements could be challenging, as concepts such as “forgiveness” and “moral values” are rather abstract and could, without sufficient examples, be hard to fully comprehend. Similarly, they warned that the idea of self-forgiveness could feel out of reach and therefore discouraging for many suffering from moral injury. Nevertheless, all assessors considered the module to be suitable for the target population and were very positive about the inclusion of videos of patient representatives. Veteran assessors, in particular, described these video examples as relatable, illuminating, and helpful.

To increase acceptability, several assessors mentioned the importance of emphasizing confidentiality. They suggested more explicitly addressing confidentiality concerns at the start of the module, by explaining how the content is protected and whether a therapist will be able to see any of the answers filled in. Some assessors suggested repeating this information when an exercise asks for self-disclosure, to increase openness and lower any distrust patients may feel.

Overall, all assessors expected the module to be beneficial to those suffering from moral injury. As potential benefits, they mentioned better insight into one's distress, easier communication about moral injury with others, and an increased understanding of how therapy may help.

#### Feasibility

Assessors generally found the module to be feasible and user-friendly. Several assessors raised questions about how to incorporate the module into existing treatments, to maximize its benefits and decrease potential risks. They suggested that synchronizing the module with face-to-face therapy, as intended, would enable patients to discuss certain topics and build on exercises with their therapist. Simultaneously, this could make it easier for patients to reach out for support if elements of the module were experienced as triggering.

#### Engagement

The information provided by the module was considered to be clear and concise. The videos were found to be particularly informative and engaging. Some assessors offered suggestions for changing certain wordings or for expanding explanations to improve comprehensibility.

Assessors felt Minddistrict was easy to use, though some commented the module could be improved by having a menu for navigating between different chapters. Several other practical suggestions for improving the module were offered, including adding various exercises.

#### Unintended consequences

Most assessors raised concern that some elements of the module could be triggering, particularly for patients experiencing a lot of distress. Potentially triggering elements of the module include the videos of patient representatives sharing their stories and a narrative writing assignment. At the same time, assessors believed that these unintended consequences would be manageable and acceptable if patients would be able to reach out to a therapist when necessary.

### Adaptation

Based on the evaluation, module content was adapted. The main adaptations were that privacy issues were explained more elaborately, safety instructions (i.e., asking patients to choose a good time and place to complete an assignment) were repeated more often, collaboration with the therapist was explained more clearly, and role play videos were shortened. In addition, psychoeducation and assessments were adapted to provide a stronger focus on moral injury due to moral transgressions by others (so-called betrayal trauma).

## Discussion

We reported on the development phase of an online treatment module for moral injury. Development resulted in an online module consisting of eight chapters and including written text, videos and assignments. The concept module was evaluated and rated favorably by various stake holders and experts. In addition, assessors commented favorably on the module's acceptability, feasibility and engagement. However, they feared that some module components including some videos and assignments might be emotionally upsetting when watched or completed at home. Module content was then adapted.

The favorable evaluation of the module is in line with other studies that show that blended treatment, combining online modules with face-to-face treatment, is perceived as purposive and effective (37). Indeed, blending may be key given that patients' motivation to engage in internet-based interventions may be relatively low. In one study of military veterans' willingness to use e-mental health, only 50.6% of those without PTSD and 30.9% of those with probable PTSD were willing to try online computer-based interventions (38). A study of internet-based TF-CBT for service members without face-to-face contact resulted in a 32.3% drop-out (39)—a percentage that is relatively high (40).

Although the module was rated favorably, its current form may have some disadvantages. While the module fits in a tradition of online CBT interventions for mental health, this tradition has been criticized for failing to stimulate adherence and sustained engagement (41). An argument has been put forward for creating digital tools that provide a better fit with patients' lives and practitioners' workflows, and involving patients and practitioners from the start of development (41). In hindsight, we indeed feel that patient representatives might have been involved more actively from the start of development and might have been asked which facets of moral injury should be addressed in the module in general and in the patient representatives' videos. As for practitioners' workflows, the module was developed in a digital platform that is widely used by practitioners to support face-to-face treatment.

Regarding unintended consequences, assessors feared that some video material and assignments might be emotionally upsetting. Indeed, in a study of internet-based TF-CBT for service members, 9.5% reported severe resistance against writing assignments, and another 23.8% experienced intense negative feelings while they were writing (38). However, another study of internet-based TF-CBT showed that adverse events and treatment satisfaction ratings were equal in two treatment arms with and without exposure components (42). Thus, although writing assignments may be perceived as emotionally challenging, this does not necessarily limit treatment satisfaction and effectiveness.

In conclusion, this module is the first online module to support face-to-face treatment of moral injury in police officers and military veterans. It was carefully developed based on development guidelines and the extant literature and in collaboration with patient representatives and experts from multiple disciplines. After evaluation, the module was adapted. In the near future, the adapted module will be evaluated in a feasibility and acceptability study using both quantitative and qualitative research methods. In addition, adapting the module to other populations, such as morally injured refugees or healthcare workers, may be considered.

## Data availability statement

The raw data supporting the conclusions of this article will be made available by the authors, without undue reservation.

## Author contributions

JH developed the module, conducted the evaluation, and drafted the first version of this paper. MG analyzed the qualitative and quantitative outcomes and drafted the first version of this paper. SD developed the module, conducted the evaluation, and contributed to the final version of this paper. SE developed the module and contributed to the final version of this paper. All authors contributed to the article and approved the submitted version.

## Funding

The development of the module was funded by a VIPP-GGZ grant from the Dutch Ministry of Public Health, Wellbeing and Sports, and by ARQ Centrum'45.

## Conflict of interest

The authors declare that the research was conducted in the absence of any commercial or financial relationships that could be construed as a potential conflict of interest.

## Publisher's note

All claims expressed in this article are solely those of the authors and do not necessarily represent those of their affiliated organizations, or those of the publisher, the editors and the reviewers. Any product that may be evaluated in this article, or claim that may be made by its manufacturer, is not guaranteed or endorsed by the publisher.
